# Supplementary figures and images for: Selecting 16S rRNA Primers for Microbiome Analysis in a Host–Microbe System: The Case of the Jellyfish Rhopilema nomadica
Source: Microorganisms. 2023 Apr 6;11(4):955. doi: 10.3390/microorganisms11040955 (PMC10144005; doi:10.3390/microorganisms11040955)

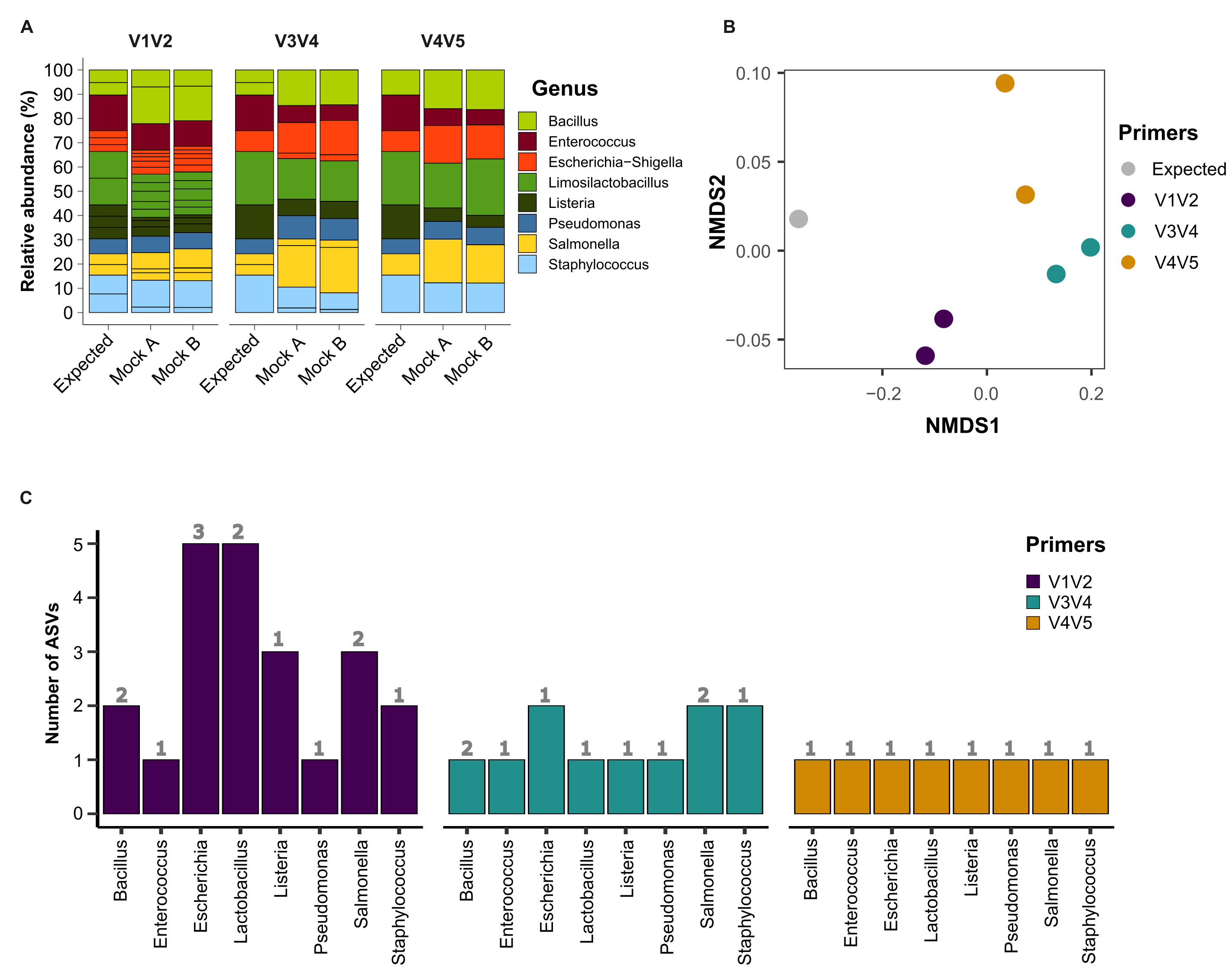

Supplement: Supplementary file 1 [file microorganisms-11-00955-s001.zip › Supplementary/Figure_S1.png]

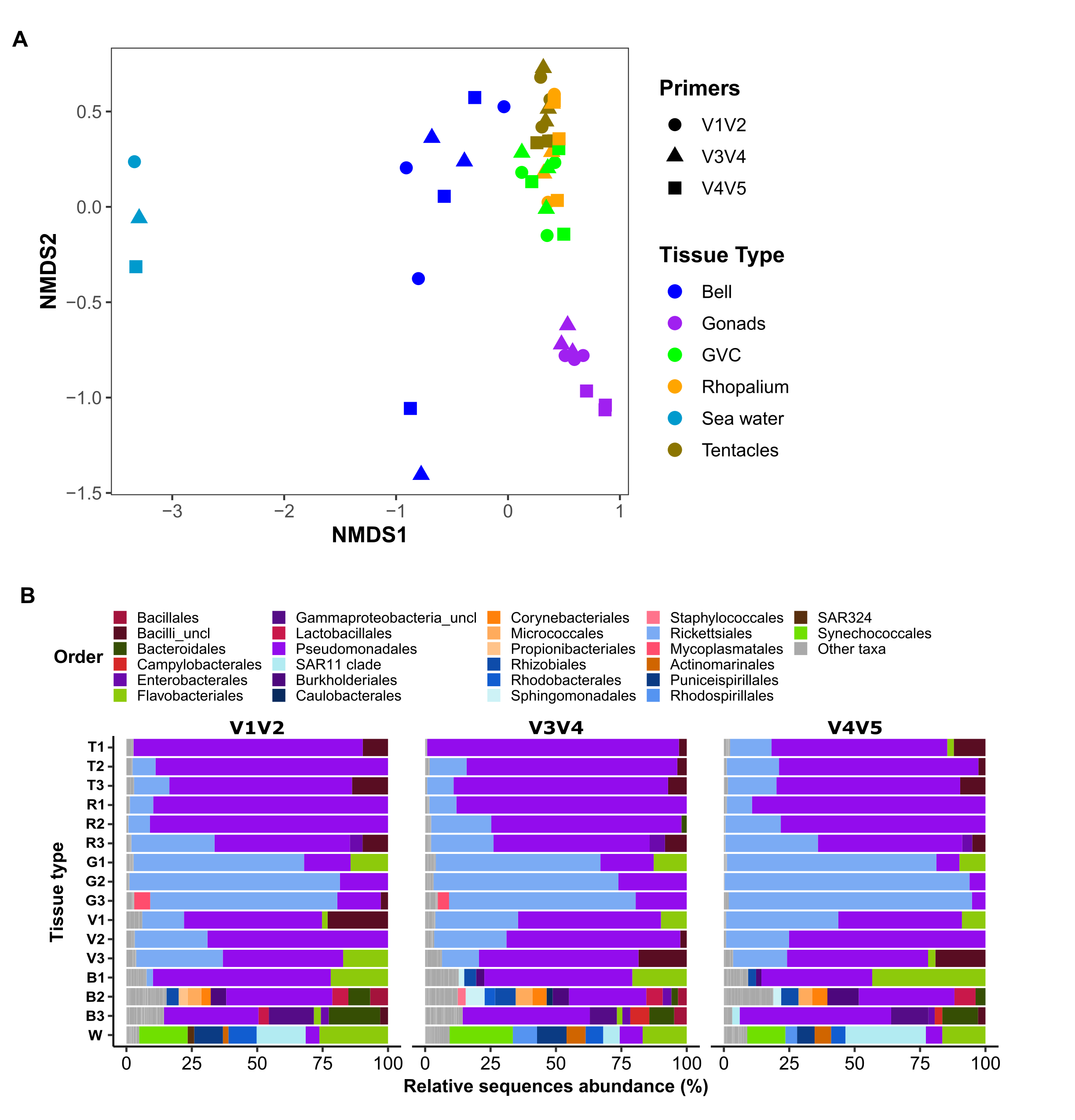

Supplement: Supplementary file 1 [file microorganisms-11-00955-s001.zip › Supplementary/Figure_S2.png]
